# Central hepatopancreatoduodenectomy—oncological effectiveness and parenchymal sparing option for diffusely spreading bile duct cancer: report of two cases

**DOI:** 10.1186/s12893-020-01012-2

**Published:** 2021-01-06

**Authors:** Kapil Nagaraj, Yuichi Goto, Satoki Kojima, Hisamune Sakai, Toru Hisaka, Yoshito Akagi, Koji Okuda

**Affiliations:** 1 Division of Hepatobiliary and Pancreatic Surgery, Department of Surgery, Kurume University School of Medicine, 67 Asahi-machi, Kurume, 8300011 Japan; 2 Department of Surgical Gastroenterology, Jawaharlal Institute of Postgraduate Medical Education and Research (JIPMER), Puducherry, India

**Keywords:** Hepatopancreatoduodenectomy, Diffusely spreading bile duct cancer, Central liver resection, Liver parenchymal sparing, Case report

## Abstract

**Background:**

Hepatopancreatoduodenectomy (HPD) for diffusely spreading bile duct cancer (DSBDC) usually involves a major hepatectomy and a concomitant pancreatoduodenectomy, and is still challenging surgery because of postoperative liver failure. The present case report demonstrated two cases of DSBDC where we could achieve successful HPD with central liver resection (CHPD) as liver parenchymal sparing surgery.

**Case presentation:**

In Case 1, endoscopic retrograde cholangiography (ERC) with multiple biopsies revealed that she had DSBDC with Bismuth-Corlette type IIIA. 3D integrated images reconstructed by contrast enhanced CT and CT with drip infusion cholecystocholangiography data revealed the right antero-ventral bile duct (RAVD) confluent to the right hepatic duct and the right antero-dorsal bile duct (RADD) independently confluent to the right posterior bile duct (RPD). Tumor extended common bile duct including intrapancreatic bile duct to the left hepatic duct and RAVD, but the RADD and RPD were spared. Because the future liver remnant (FLR) was assumed not to achieve desirable volume by preoperative portal vein embolization for left or right trisegmentectomy, CHPD including resection of the segments IV and I, and the right antero-ventral segment was done and achieved R0. This procedure is tailored to the anatomical extent of disease in the context of variable biliary anatomy as a modified CHPD, and to our knowledge, this is the first reported case of modified CHPD with antero-dorsal segment preservation. In Case 2, preoperative imaging revealed DSBDC with Bismuth Corlette type IIIA. FLR volume was assumed insufficient for major hepatectomy, CHPD including resection of the segments IV and I, and the right anterior sector was done with R0. The remnant liver volumes of these cases were spared by 55.1% and 25% respectively, and postoperative course was uneventful in both.

**Conclusion:**

CHPD should be considered a valid option for well-selected cases of DSBDC. This is the first case report of modified CHPD with antero-dorsal segment preservation.

## Background

Hepatopancreatoduodenectomy (HPD) as a surgery specifically for the diffusely spreading bile duct cancer (DSBDC) usually involves a major hepatectomy, caudate lobe resection and a concomitant pancreatoduodenectomy (PD). HPD is initially introduced by Takasaki et al. [1] in 1980 for gallbladder cancer and later widely applied to hilar cholangiocarcinoma, but combination of major hepatectomy and PD increases the risks associated with the surgery exponentially. It has with time achieved better results in term of morbidity and mortality in high volume centers, however, is still challenging surgery [2].

Liver failure due to inadequate remnant liver volume remains the crucial factor for increase in postoperative morbidity and mortality. In achieving better results, the preoperative portal vein embolization (PVE) has been a valuable tool in increasing the future liver remnant (FLR) volume. In spite of this technique, not all patients achieve a desirable remnant liver volume and are often deferred from surgery. When the waiting period following PVE usually extends for 3–4 weeks, there is a certain subset of patients who have tumor progression, hence, deferred from resection. In these situations, if hepatectomy is tailored to the anatomical extent of disease in the context of variable biliary anatomy, parenchyma sparing HPD instead of merely extended hepatic resections based on the sidedness of the tumor may applicable as an alternative procedure to achieve R0 resection in well-selected cases.

Herewith we present two cases of DSBDC where we could achieve oncological negative margins in HPD with central liver resection (CHPD) as liver parenchyma sparing surgery [3], and desirable short term outcomes.

## Case presentation

### Case 1

**Fig. 1:**
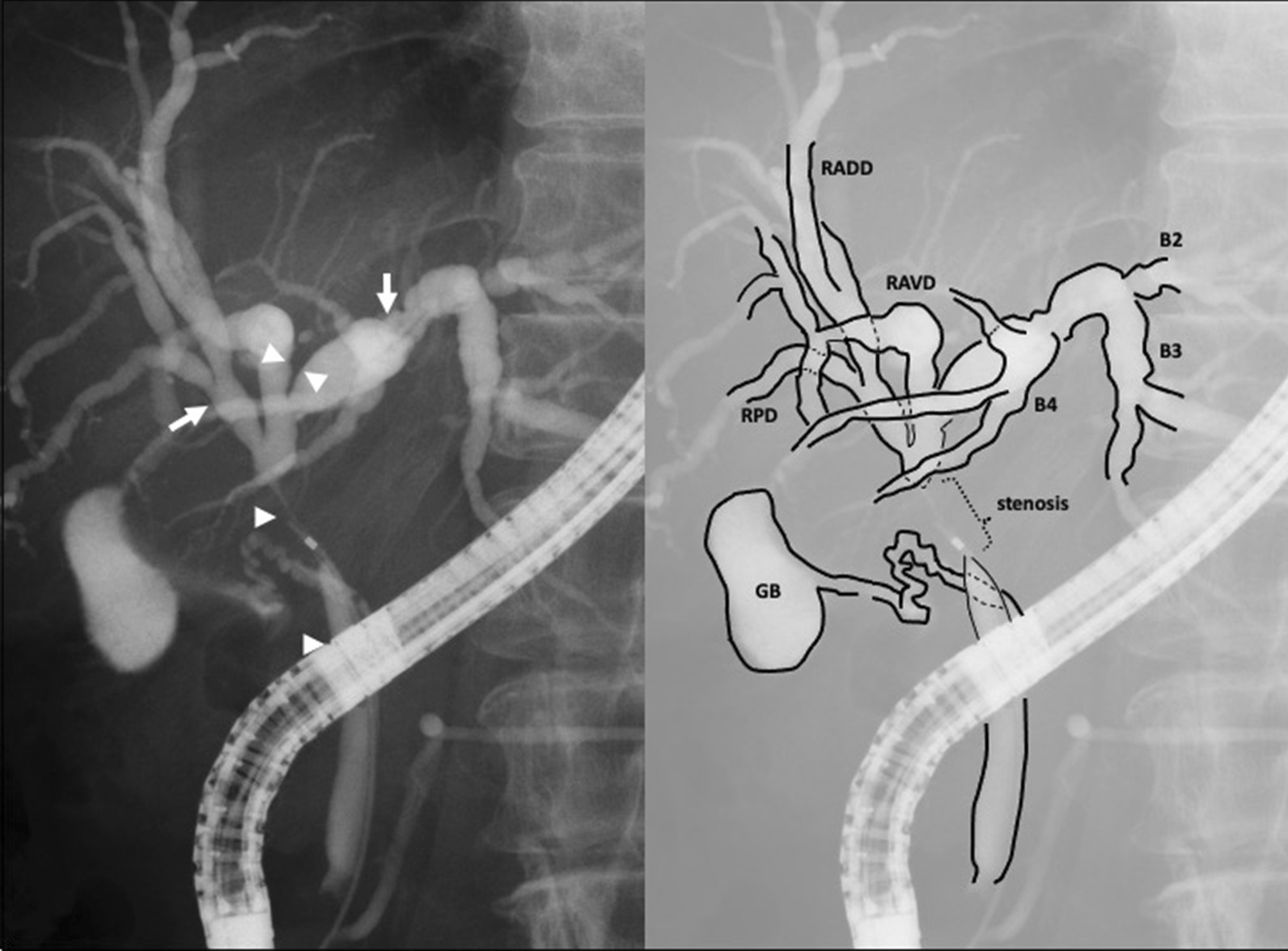
ERC image showing tissue sampling of the case 1. Arrows show the negative sampling biopsy of the right posterior bile duct and B2 + B3. Arrow heads show adenocarcinoma of the right antero-ventral bile duct, left hepatic duct, and common bile duct including intrapancreatic bile duct. *RAVD* the right antero-ventral bile duct. *RADD* the right antero-dorsal bile duct. *RPD* the right posterior bile duct, *B2* the segment II bile duct, *B3* the segment III bile duct, *B4* the segment IV bile duct, *GB* gall bladder

**Fig. 2:**
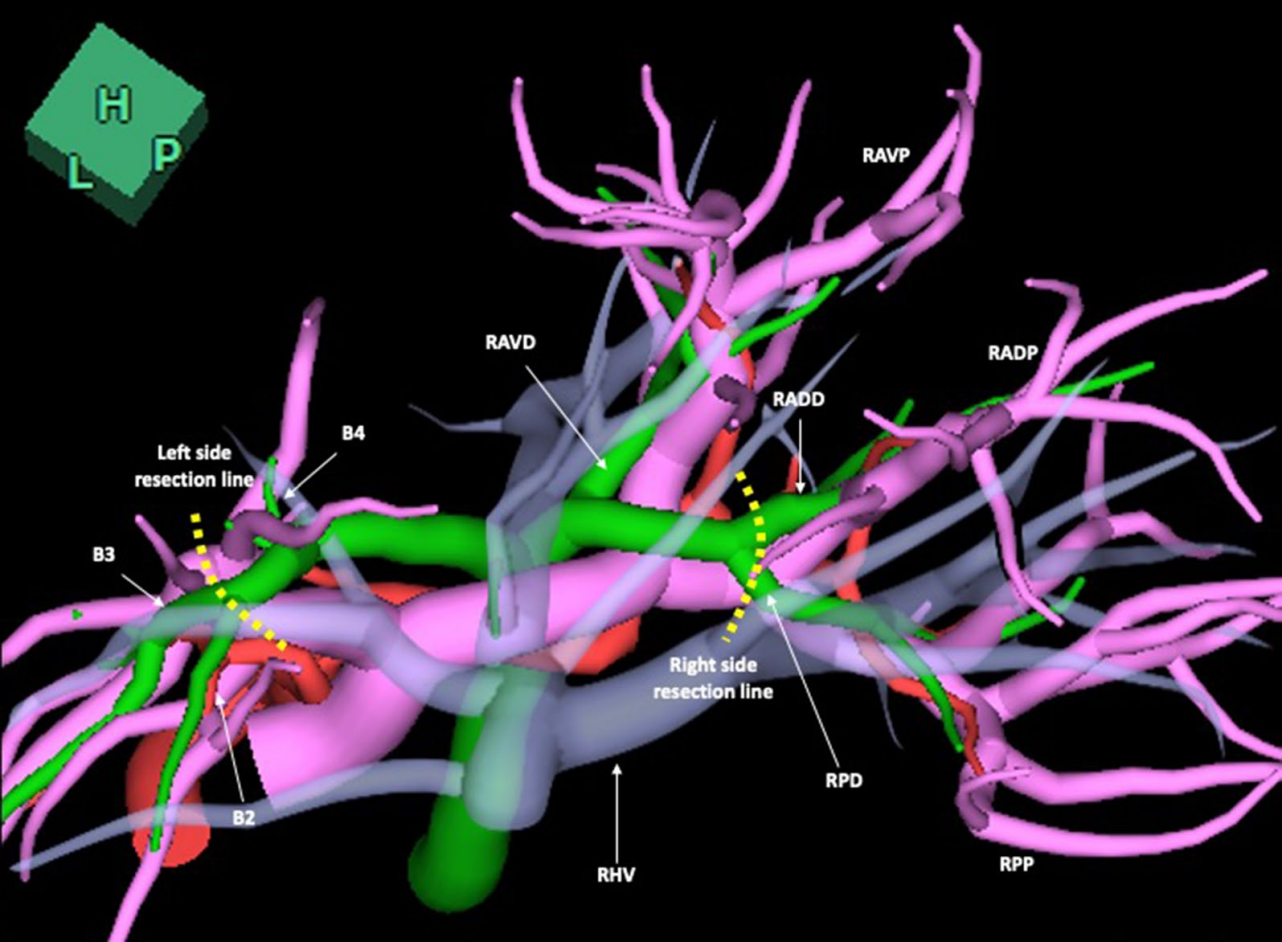
Cranial-dorsal view of the integrated 3D images of vessels (surface rendering). The right antero-ventral and antero-dorsal portal vein were independently configurated. The right antero-ventral bile duct confluent to the right hepatic duct and the antero-dorsal bile duct independently confluent to the right posterior bile duct. The broken lines show the transection of the right antero-dorsal bile duct and right posterior bile duct, also of the common duct for the segment II and III. *RAVD* the right antero-ventral bile duct, *RADD* the right antero-dorsal bile duct, *RAVP* the right antero-ventral portal vein, *RADP* the right antero-dorsal bile duct, *RPP* the right posterior portal vein, *RHV* the right hepatic vein

A 66-year-old female presented with easy fatigability and jaundice. Investigations at admission revealed a serum bilirubin of 6.8 mg/dl with no evidence of cholangitis. Clinical variables have been delineated in Table 1. Contrast enhanced CT (CECT) and magnetic resonance cholangiopancreatography (MRCP) showed an irregular enhancing wall thickening of the sub hilar region extending distally. She had preoperative biliary drainage by endoscopic nasobiliary drainage (ENBD). Endoscopic retrograde cholangiography (ERC) with multiple biopsies for tumor mapping revealed that the adenocarcinoma of common bile duct including intrapancreatic bile duct that was extended to the left hepatic duct (LHD) (Fig. 1). Hence, tumor was designated as DSBDC with Bismuth-Corlette type IIIA, which involved the right anterior bile duct (RAD) and junction of the right posterior bile duct (RPD) and the LHD, and extended to the intrapancreatic bile duct. Once her serum bilirubin dropped to < 3 mg/dl, CT with drip infusion cholecystocholangiography (DIC-CT) was done. 3D integrated visualization reconstructed by CECT and DIC-CT data revealed that she had independent configuration of the right antero-ventral portal branch and antero-dorsal portal branch (Fig. 2) [4]. The right antero-ventral bile duct (RAVD) confluent to the right hepatic duct (RHD) and the right antero-dorsal bile duct (RADD) independently confluent to RPD with a supra-portal course. Therefore, images showed tumor extended to the RAVD, but the RADD was intact because it was confluent to the distal side of the RPD. A modified central hepatectomy including resection of the segments IV and I, and right antero-ventral segment was done concomitantly with PD, and R0 resection was achieved. CT volumetry findings have been discussed in Table 2. Postoperative functional assessment of liver is included in Table 1. She underwent staged pancreato-jejunostomy 6 months later. She is doing well without recurrence at 17 postoperative months.

**Table 1:** Characteristics of patients and laboratory findings

Variables	Case 1	Case 2
Age (years)	66	67
Sex	female	male
BMI (kg/m^2^)	22.0	23.8
ECOG Performance Status	0	0
Serum bilirubin level at admission (mg/dL)	6.8	14.9
AST (IU/L)	27	268
ALT (IU/L)	25	514
Biliary anatomy	Right antero-ventral bile duct confluent to the right hepatic duct and the right antero-dorsal bile duct independently confluent to the right posterior bile duct	Right posterior bile duct draining into the left hepatic duct
Bismuth-Corlette type	IIIA	IIIA
Serum bilirubin level a day prior to resection (mg/dL)	0.8	1.6
Cumulative Pringle time in operation (min)	62	75
Operative blood loss (mL)	291	890
Total operation time (min)	1158	970
Serum bilirubin (POD 5) (mg/dL)	1.8	1.1
Serum AST/ ALT (POD 5) (mg/dL)	39/137	16/66
PT-INR (POD 5)	0.94	1.0
Clavien-Dindo grading of morbidity	Nil	Grade II—POPF
Staging AJCC (8th edition)	BpBd, flat-infiltrating type, circ, 1.2 × 1.0 × 3.5 cm, pT2, por2, ly(0), v1, ne1, pN0, pDM0, pHM0, pEM0, pPV0, pA0, R0, M0 Stage II	BpBd, papillary invasive type, circ, 1.2 × 0.6 × 7.0 cm, pT1, pap, ly0, v0, ne0, pN0, pDM0, pHM0, pEM0, pPV0, pA0, R0, M0 Stage I

*BMI* body mass index, *ECOG* Eastern Cooperative Oncology Group, *AST* asparate aminotransferase, *ALT* alanine aminotransferase, *POD* post operative day, *PT-INR* prothrombin time international normalized ratio, *POPF* post operative pancreatic fistula, *AJCC* American Joint Committee on Cancer

**Table 2:** Volumetry data and functional assessment of liver

Case	TLV (ml)	FLR in left trisegmentectomy (ml/% of TLV)	FLR in right trisegmentectomy (ml/% of TLV)	FLR in central hepatectomy (ml/% of TLV)	Parenchymal sparing achieved (ml/% of TLV)
1	1055	172/16.3%	193/18.2%	764/72.4%	581/55.1%
2	1629	499/30.7%	318/19.5%	817/50.2%	408/25.0%

*TLV* total liver volume, *FLR* future liver remnant

### Case 2

**Fig. 3:**
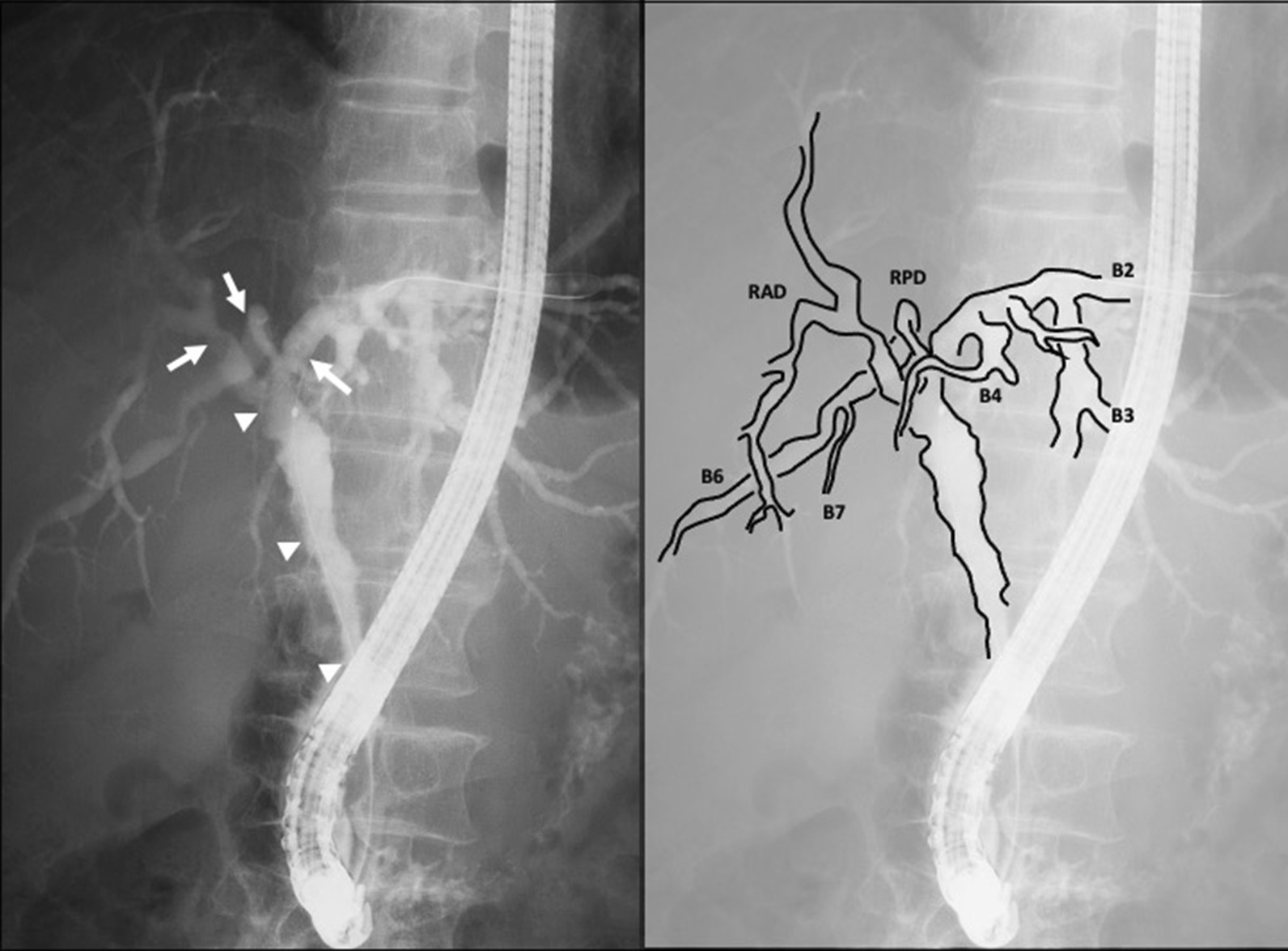
ERC image shows the right posterior bile duct join to the left hepatic duct. Arrows show biopsy points, and biopsies revealed that high grade dysplasia extending to the left hepatic duct, right anterior duct and root of right posterior duct. However, ERC image shows the distal side of the right posterior duct is not involved. Arrow heads revealed adenocarcinoma of hepatic hilum to the intrapancreatic bile duct. *RAD* the right anterior bile duct, *RPD* the right posterior bile duct, *B2* the segment II bile duct, *B3* the segment III bile duct, *B4* the segment IV bile duct, *B6* the segment VI bile duct, *B7* the segment VII bile duct

**Fig. 4:**
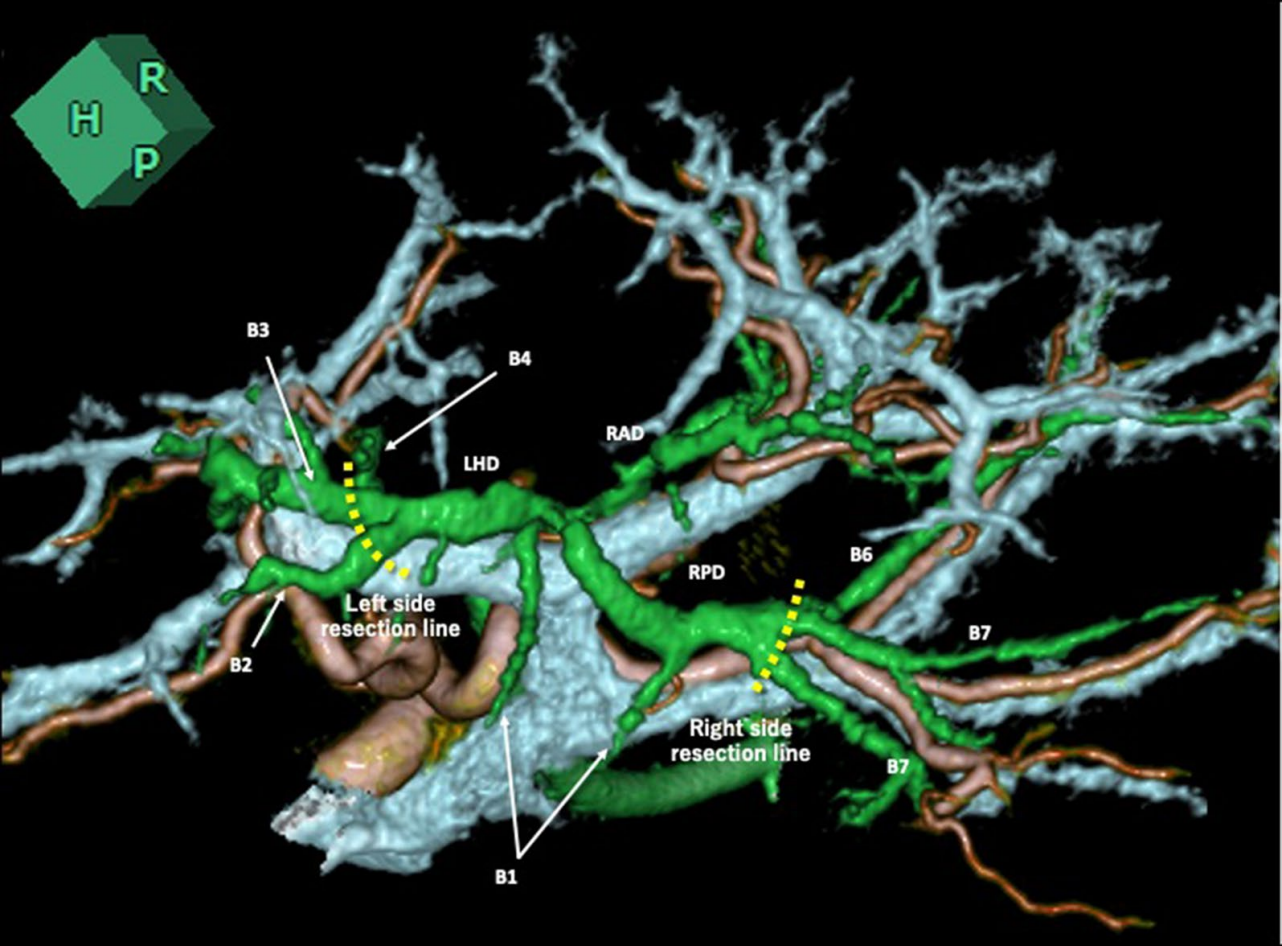
Cranial-dorsal view of the integrated 3D images of artery, portal vein and bile duct (volume rendering). The right posterior portal vein branches independently from main portal vein. Supraportal course of the right posterior bile duct is seen. The broken lines show transection of the third order segmental bile ducts for segments VI and VII, and for segment II and III. *RAD* the right anterior bile duct, *RPD* the right posterior bile duct, *LHD* the left hepatic duct, *B1, B2, B3, B4, B6 and B7* the third order bile ducts for the segments I, II, III, IV, VI and VII

A 67-year-old male presented with jaundice. Investigations revealed a serum bilirubin of 12.4 mg/dl with evidence of cholangitis. Clinical variables have been delineated in Table 1. CECT showed an irregular wall thickening of the distal bile duct region which extended proximally. MRCP suggested distal bile duct stricture with dilatation proximally. He had preoperative biliary drainage by ENBD, and endoscopic tumor mapping using multiple targeted biopsies revealed adenocarcinoma extending intrapancreatic bile duct to the configuration of right and left hepatic ducts. Additionally, high grade dysplasia was evident from the LHD, RAD and root of RPD. Hence, tumor was designated as DSBDC with Bismuth-Corlette type IIIA hilar component. However, ERC shows the distal side of the RPD was not involved (Fig. 3). 3D integrated images using CECT and DIC-CT data revealed that RPD join to LHD with supraportal course (Fig. 4). CT volumetry findings have been discussed in Table 2. CHPD, which consist of the segments IV and I, and the right anterior sector, was performed with R0 resection. Postoperative liver functional assessment and pathological findings of resected specimens have been included in Table 1. He underwent staged pancreato-jejunostomy 4 months later, and doing well without recurrence at 19 postoperative months.

## Operative procedures

**Fig. 5:**
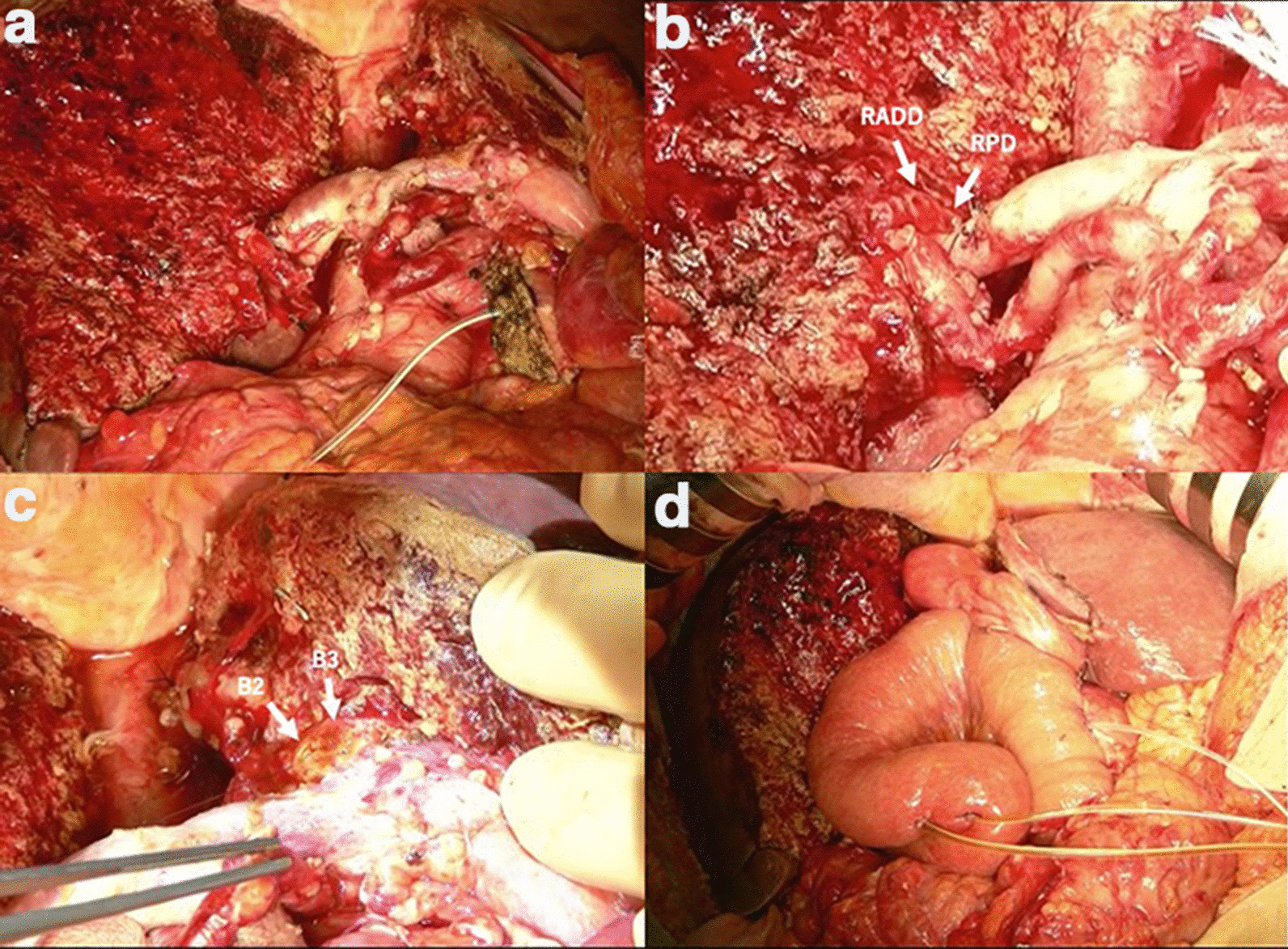
Intraoperative images after bile duct transection and resected specimen retrieved in the case 1 (**a**). These images show the limits of resection of central HPD. The image **b** shows the divided ends of RADD and RPD (arrows). The image **c** shows the left side resection limit—divided ends of ducts B2 and B3 to the left of umbilical fissure (arrows). The image **d** shows after reconstruction. *RADD* the right antero-dorsal bile duct, *RPD* the right posterior bile duct

A standard central hepatectomy including resection of the segments IV and I, and the right anterior sector in case 2 and a modified central hepatectomy including resection of the segments IV and I, and the right antero-ventral segment in case 1 were done concomitantly with PD. PD was done first proceeding cranially with lymphadenectomy along the hepatoduodenal ligament simultaneously skeletonizing the vascular structures. Preparation and isolation of the right and left hepatic artery, and dissection of the middle hepatic artery were done followed by dissection of the right anterior sectoral branch (in case 2) or the right antero-ventral segmental branch (in case 1) at its origin. The left portal vein and right portal vein were isolated and small branches to the segments I and IV were dissected meticulously. The branch for the right anterior sector (in case 2) or the right antero-ventral segment (in case 1) was dissected to be looped. Complete mobilization of liver including the caudate lobe was accomplished. Arantius ligament was divided and with traction over it the LHV-MHV junction was looped by a silicon tape for a modified hanging technique [5]. Under intermittent hepatic inflow occlusion, liver transection was begun at the umbilical fissure level and proceeded towards the left hepatic vein. Meanwhile after the left hepatic vein was visualized, the transection plane was further determined using traction of the silicon tape which encompass Spiegel lobe. Finally, the bile duct was left intact for transection at a later stage. Subsequent transection was along the interface between the right anterior and right posterior sector along the right hepatic vein in case 2. In case 1 parenchymal transection was proceeded along the anterior portal fissure [4,6], which was demarcated as an ischemic area. In the deeper plane, the silicon hanging tape was re-positioned between the segment VI and the right caudate process to remove the right caudate lobe. At the final stages, transection of the third order segmental bile ducts was done and the specimen retrieved (Fig. 5). Reconstruction was by Roux-en-Y cholangiojejunostomy using 6-0 PDS; interno-externally stented. We chose external drainage of pancreatic duct and staged pancreatic reconstruction. Second stage pancreaticojejunostomy was done after 3 months.

## Discussion

HPD is often associated with high morbidity (20–66%) and mortality (0–9%) even with high volume centers [7]. Integral strategy to offer consistency has been well planned operation using CECT with three dimensional reconstruction including volumetry, extensive endoscopic assessment for tumor mapping and preemptive measures such as biliary drainage, PVE and staged pancreatic reconstruction to reduce the morbidity and thereby mortality [8].

Liver failure remains the most common cause of postoperative morbidity [9]. PVE has become an essential technique to induce preoperative hypertrophy of FLR [10,11]. In isolated liver resection, usually target liver remnant volume is more than 25% to total liver volume percentage in patients with a normal liver, or up to > 30–40% in patients with cholestasis or suspected poor liver quality [7]. With combination of PD, the remnant volume should be higher than the isolated liver resection criteria. Therefore, in spite of preoperative PVE, not all patients achieve a desirable FLR volume and some patients has rapid tumor progression in waiting period after PVE, and those are deferred from surgery. Recently, associating liver partition and portal vein ligation surgery (ALPPS) has been introduced to obtain a rapid volume enhancement of the FLR. However, a morbidity after ALPPS was reported to be high, and the mortality rate was 9–15% of patients even in cases of isolated liver resection [12–14]. ALPPS remains out of question to date when it comes to planning HPD [15]. Hence, the parenchyma sparing technique in HPD are seen as a viable alternative.

CHPD is by definition resection of right anterior sector and caudate lobe with or without resection of segment IV along with extrahepatic bile duct and pancreatoduodenal complex. This is a parenchyma sparing procedure to preserve the remnant liver volume and very few cases have been reported in literature [16–19]. Median remnant liver volume increased by 31.2% when the procedure was modified to a central hepatectomy in a series by Mizuno et al. [17]. In a meta-analysis by Li et al. [20], they compared extended hepatectomy and mesohepatectomy; concluded that the liver failure was less common in the mesohepatectomy group. In our cases, the mean remnant liver volume increased by a 40% and none of them had post-operative liver failure as defined by ISGLS [21].

While Bismuth-Corlette type II, type III and well selected type IV hilar cholangiocarcinoma are accepted indications for CHPD, technically demanding nature of the procedure and the evidence for higher risk of positive margins from limited case series reduced its applicability [7,17]. Drawbacks such as the need for limited circumferential spread, procedure being prolonged in nature and technically demanding have been discussed in literature [17]. Prolonged duration is attributed to the increase in transection surface area and the difficult dual biliary anastomosis, however, they did not reflect in the short-term outcomes. As far as margins are concerned, given the supraportal course of RPD being the most common in occurrence, the additional length of about 6–9 mm gained by combining the resection of the right anterior portal vein in central hepatectomy is often less considered [7,22]. This technical point is similarly applied in offering left trisectionectomy over left hepatectomy to improve marginal clearance [23]. Similarly, the dividing point on the left is to the left of umbilical fissure which is the limit of resection when it comes to right trisectionectomy. Extending the proximal dividing point just proximal to the third order branching is possible with meticulous parenchymal dissection and segmental artery preservation, thereby providing improved marginal clearance. Further, bile duct anatomy is variable at hepatic hilum. Segment IV duct converging into LHD close to hilum and a trifurcation of hilar bile ducts with absent RHD are typical examples of anatomical variants in which the sectorial ducts are often involved very early; designating them as locally advanced and proposing extended resection in these cases may be unnecessary. The preoperative accurate anatomical understanding of the hilar vessels and appropriate operative planning is essential in achieving a successful resection. In our series, 3D integrated images of hepatic artery, portal vein, hepatic vein and bile duct, which were imported from CECT and DIC-CT data were applied in preoperative planning of resection. In one of our case, distinct ventro-dorsal distribution of biliary and portal venous anatomy further invoked a strategy of modification of the central hepatectomy, which involved a resection of antero-ventral segment, left medial segment and caudate lobe, to spare much more parenchyma [19]. To our knowledge, this is the first reported case of modified CHPD with antero-dorsal segment preservation.

Selecting patients for upfront CHPD will always be a difficult decision in the era of preoperative PVE and its proven benefits. Major hepatectomy is first considered in principle, however, we sometimes encounter patients who cannot undergo HPD with major hepatectomy because of a poor hepatic reserve or the small FLR volume expected before PVE. FLR volume is calculated assuming that FLR would gain 10% in terms of the entire liver volume after PVE [24,25]. For these cases upfront CHPD is applicable as an alternative procedure. Further, for those who have failed to achieve adequate liver remnant functional recovery after PVE, CHPD remains the only viable option provided the chances of achieving a negative margin. CHPD as a secondary option should be overlooked although it has inherent issues like the questionable functional recovery of the embolized lobe spared for want of volume and higher risk of positive margins by virtue of their selection for extended resections as the primary option.

## Conclusion

We are hereby proposing that CHPD will be a valid option for well-selected cases of DSBDC, and reported the first case of modified CHPD with antero-dorsal segment preservation. Ability to achieve negative oncological margins and at the same time preserve liver parenchyma are the key components of this surgery. Further scrutiny of its anatomical and oncological applicability and long-term prognosis is needed.

## Data Availability

All the data supporting our findings is contained within the manuscript.

## Abbreviations

ALPPS: Associating liver partition and portal vein ligation surgery
CECT: Contrast enhanced CT
CHPD: Hepatopancreatoduodenectomy with central liver resection
DIC-CT: CT with drip infusion cholecystocholangiography
DSDBC: Diffusely spreading bile duct cancer
ENBD: Endoscopic nasobiliary drainage
ERC: Endoscopic retrograde cholangiography
FLR: Future liver remnant
HPD: Hepatopancreatoduodenectomy
LHD: Left hepatic duct
LHV: Left hepatic vein
MHV: Middle hepatic vein
MRCP: Magnetic resonance cholangiopancreatography
PD: Pancreatoduodenectomy
PVE: Portal vein embolization
RAD: Right anterior bile duct
RADD: Right antero-dorsal bile duct
RAVD: Right antero-ventral bile duct
RHD: Right hepatic duct
RPD: Right posterior bile duct
